# Spatial nitrifications of microbial processes during composting of swine, cow and chicken manure

**DOI:** 10.1038/srep14932

**Published:** 2015-10-07

**Authors:** Ke Wang, Weiguang Li, Xiangkun Li, Nanqi Ren

**Affiliations:** 1 School of Municipal and Environmental Engineering, State Key Laboratory of Urban Water Resource and Environment (SKLUWER), Harbin Institute of Technology, 73 Huanghe road, Harbin, Heilongjiang 150090, China

## Abstract

Composting is a widely-used method to recycle the nutrients in livestock manure for agriculture. The spatial stratifications of microbial processes inside the manure particle that determine organic and nitrogen transformation are virtually unclear. Here, we show the evolution of the interior microenvironment of swine, cow and chicken manure by using microelectrodes during forced-aeration composting. Composting has generally been regarded as an aerobic bioprocess, however, the long-existing of a large anoxic zone inside these manures was confirmed during the active phase in this study. The profile of the oxidation–reduction potential dramatically decreased first and then gradually increased. The spatial difference in the ammonia concentration was not significant, but nitrate concentration continuously decreased with depth. The anoxic condition within the manure particle was demonstrated to be a primary cause of the severe ammonia emission and the long composting period. These founding provided a new insight toward “aerobic” composting process and a sound foundation for the development of efficient composting technology.

Livestock waste is an important environmental pollution source as well as a valuable nutrient resource^1^. Aerobic composting is a traditional method of biologically transforming livestock feces to organic fertilizer in the presence of oxygen^2^. Forced aeration, oxygen concentration control and a large amount of bulking agents are used in the commercial composting processes to provide adequate oxygen and free airspace in this solid incubation matrix^3^,^4^,^5^,^6^. Although the microenvironment in the interior of the livestock manure particles is unclear, the composting is commonly regarded as an aerobic bioprocess, and is called aerobic composting^7^,^8^.

Improving the efficiency of organic stabilizing is an important goal in the composting operations^9^. The composting process could be divided into two stages: the active stage (or the thermophilic stage) and the curing stage. Generally, these two stages require a period of more than two months period before reaching biological stabilization of the product^10^ and this long treatment period essentially impacts the application of this technology. To reduce the composting period, a lot of research has focused on optimizing incubation conditions such as temperature, aeration rate, C/N ratio, moisture, porosity, pH, adding bulking agent and turning frequency to provide the optimal environment for microbial metabolism^11^,^12^,^13^,^14^,^15^. However, the composting period has not been reduced over the last three decades.

Severe nitrogen loss is another bottleneck for the composting treatment of the livestock manure^16^,^17^. A large amount of NH_3_ released from composting pile during the thermophilic period, which not only reduces the fertilizer effect but also causes acidification and eutrophication. The low C/N ratio of livestock manure is regarded as the main reason leading to the nitrogen loss in the literature^18^,^19^. Thus, a large amount of carbon-rich bulking agents, such as woodchips, straw and sawdust, is input into the feedstock before the livestock manure composting^20^,^21^,^22^,^23^,^24^. Although the consumption of the bulking agents greatly increases the operational cost, the issue of nitrogen loss has not been well resolved. It was reported that the ammonification and nitrification of microorganisms play important roles on the nitrogen loss in the composting matrix^25^,^26^,^27^,^28^. However, the manure particle is the basic unit of the composting matrix, and the spatial distribution characteristics of microbial nitrogen transformation in the interior of manure particle have not been understood.

Oxygen level not only affects the bio-stabilization rate of the organics but also the transformation process of the nitrogen in the composting process^29^,^30^,^31^,^32^. However, electron acceptors (oxygen) and donors (soluble organic matter and reduced nitrogen) are subject to mass transfer limitations. Oxygen transfer process in the composting matrix can be divided into the interparticle stage (the space between particles) and the interior particle stage (inside the particle)^6^. The bulking agent provides the structural support to create interparticle voids that act as the air-flow channels. However, the spatial stratifications of the microbial activity, dissolved oxygen (DO) and chemical changes (e.g., 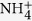 and 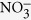 concentration) in the interior of the manure particle are not clear. This information is quite necessary for an understanding of the process of nitrogen transformation and organic biodegradation in composting system.

In this study, oxygen transfer characteristics and spatial distribution of microbial nitrogen transformation inside chicken, cow and swine manures were investigated during composting. The chemical gradients of DO, oxidation–reduction potential (ORP), and 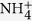 and 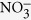 concentration were determined for the first time in the surface layer of three manures using microelectrodes during the composting period. The effects of oxygen permeation depth, ORP, C/N, pH, dissolved organic carbon, temperature and moisture on NH_3_, CO_2_ and CH_4_ emission rate of the three manures were evaluated. The relationships between oxygen transfer and the manure stabilization process and between microbial stratification and chemical gradients were elucidated.

## Results

### 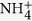 in the micro-profile of manure particles

**Figure 1:**
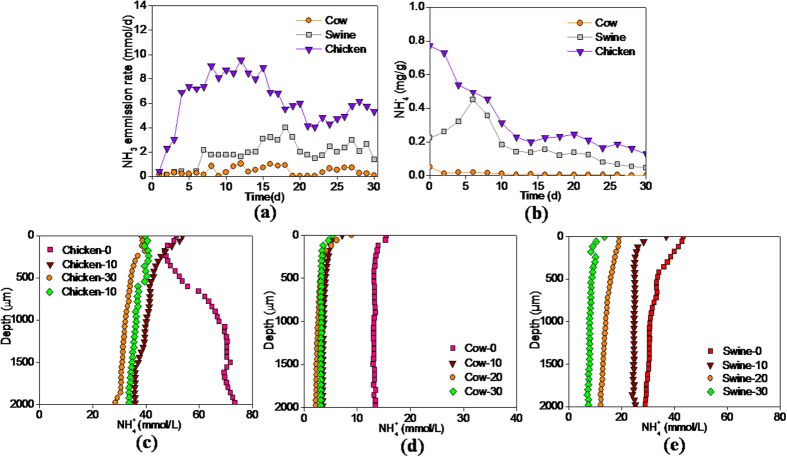
Variation of (**a**) NH_3_ emission rate and (**b**) 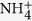/NH_3_ content of the swine, cow, chicken manures during composting; Variation of 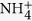
concentration with depth in the microprofile of the manure particles of chicken (**c**), cow (**d**) and swine (**e**) on Day-0, Day-10, Day-20 and Day-30.

The C/N ratios in the raw swine and chicken manure (Day-0) were closed (8.0 and 8.3), but their difference in NH_3_ emission rate was noticeable. The cumulative amount of the NH_3_ emission from the chicken manure (189.4 mmol) was substantially higher than that from the swine manure (59.2 mmol) and from the cow manure (12.3 mmol) during 30 days of composting (Fig. 1a). This result was consistent with the order of the 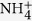
**/** NH_3_ content in the three manures (Fig. 1b). The spatial difference in the 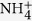 concentration in the interior of the manure particles was not significant, which was in-site detected by 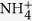
microelectrode (Fig. 1c–e). This finding may be attributed to the dynamic balance of NH_3_ volatilization and re-absorption in the liquid phase.

Although a mass of 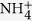/NH_3_ was released into the liquid phase from biological ammonification^25^,^33^, the 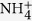/NH_3_ content was synchronously reduced in the three manures as NH_3_ emission during the initial period. The ammonification also increased the pH value of the three manures. The pH value increased from 8.1 to 9.0 in the swine manure, from 8.0 to 8.6 in the cow manure, and from 8.4 to 9.4 in the chicken manure during the first 10 days, respectively. The increase in the pH value significantly promoted the transformation from 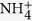 to NH_3_ in the 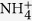/NH_3_ pool, and improved NH_3_ emission^24^,^25^,^26^,^27^,^28^. Furthermore, the moisture reduced by 59.1% in the swine manure, 51.7% in the cow manure and 64.9% in the chicken manure during the first 10 days. Meanwhile, the solubility of NH_3_ in water decreased from 520 to 200 g NH_3_ (kg water)^−1^ as the incubation temperature rose from 20 to 55 °C. Due to the moisture loss and temperature decrease, the maximum NH_3_ dissolving capacity was reduced by 84.3% 81.4% and 86.5% in the swine, cow and chicken manures during the first 10 days, respectively. As a result, the 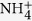/NH_3_ content dropped from 771.6 to 312.1 mg kg^−1^ in the chicken manure, from 450.8 to 184.5 mg kg^−1^ in the swine manure and from 10.1 to 6.4 mg kg^−1^ in the cow manure. Therefore, ammonification, moisture loss and temperature increase are important causes of the severe NH_3_ emissions.

### CO_2_ and CH_4_

**Figure 2:**
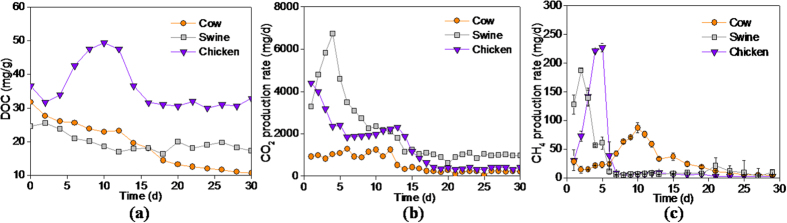
Variation of dissolved organic carbon content (**a**), CO_2_ emission rate (**b**) and CH_4_ emission rate (**c**) of the swine, cow, chicken manures during composting.

Dissolved organic matters are the direct carbon sources for the microbes in the composting matrix. The content of dissolved organic carbon in the chicken manure (47.6 to 30.1 mg g^−1^) was consistently higher than that in the swine manure (29.7 to 16.3 mg g^−1^) and the cow manure (31.7 to 10.6 mg g^−1^) during the entire test (Fig. 2a). The significant decline in the dissolved organic carbon content was mainly ascribed to rapid biodegradation of dissolved organic matters in the three manures. The content of dissolved organic carbon has a negative correlation with the biological stability of the compost^34^. This means that the cow manure had the highest stability among the three manures on Day-30.

The evolution of CO_2_ emission rate showed that the mineralization rate of the three livestock manures significantly decreased with composting time (Fig. 2b). The CO_2_ production rate was reduced by 90.4% for the swine manure, 85.6% for the chicken manure and 81.6% for the chicken manure during the first 15 days. This result could be related to the decrease in available organic content and the moisture. The cumulative amount of CO_2_ emissions was 62.0 g, 41.9 g and 16.9 g for the swine, chicken and cow manure within the entire test, respectively.

CH_4_ emission from the three animals’ manures was mainly occurred in the initial period of composting (Fig. 2c). This result was also supported by the previous literatures^35^,^36^. Although the swine manure had the highest CO_2_ emission rate among the three animals’ manures, the chicken manure had the highest CH_4_ emission rate during the initial period. The peak values of CH_4_ emission rate were presented on Day-2 (187.2 mg d^−1^), Day-5 (226.9 mg d^−1^) and Day-10 (87.5 mg d^−1^) for the swine, chicken and cow manures, respectively. The cumulative amounts of CH_4_ emissions were 819.0 mg, 711.1 mg and 676.7 mg for the swine, chicken and cow manures during the incubation, respectively. Because the methane is produced in a strictly anaerobic environment, the methane generated from the three manures indicated the existing of the anaerobic environment inside the three animals’ manures.

### DO in the micro-profile of manure particles

**Figure 3:**
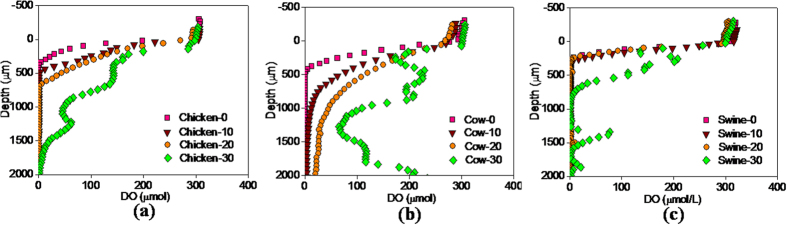
Variation of DO with depth in the microprofiles of the manure particles of chicken (**a**), cow (**b**) and swine (**c**) on Day-0, Day-10, Day-20 and Day-30.

The depth profiles of the DO concentration in the surface layer of the three manures were measured by the DO microelectrode on Day-0, Day-10, Day-20 and Day-30 (Fig. 3). The DO concentrations began to slowly decrease at 0.4–0.3 mm above the surface of the manure particles. This was probably caused by resistance to mass transfer or the diluting effects of water vapor releasing from the manure. The DO concentration rapidly dropped with the depth in the surface layer of the three raw manures on Day-0. The DO was depleted in the top 0.30 mm of raw swine manure, 0.42 mm of raw chicken manure and 0.50 mm of raw cow manure, and the rest of the raw manure particles were anoxic. After 20 days forced-aeration composting, oxygen penetrated 0.78 mm in the chicken manure and 2.10 mm in the cow manure on Day-20, whereas the oxygen penetration depth in the swine manure still remained approximate 0.30 mm (Fig. 3b). These findings indicate that the aerobic zone was consistently limited to the surface layer of the three manure particles over the active stage. Oxygen diffusion in the manure particles appears to be the critical rate-limiting step for the oxygen transfer process in the composting matrix. The average diameter of the manure particles was measured to be larger than 8 mm on Day-20, indicating more than 75% of the swine manure particle, 50% of the chicken manure particle and 10% of the cow manure particle was the anoxic zone. Composting has generally been regarded as an aerobic bioprocess. However, this work confirmed that a significant volume of anoxic zone existed in the interior of the three manures during the active stage.

The DO gradient inside the manure particles mainly depends on the balance between the oxygen diffusion rate and microbial oxygen utilization rate. The close depths of oxygen permeation in the three raw manures suggested their closed value of oxygen utilization rate at the beginning of composting. According to the Fick’s first law^37^, the average oxygen flux in the aerobic layer was 0.556, 0.214 and 0.140 μmol (cm^2^·h)^−1^ in the swine, chicken and cow manure on Day-20, respectively. The oxygen utilization rate in the surface of the swine and chicken manure was significantly higher than that of the cow manure. This result was consistent with the order of their CO_2_ production rate. The differences in oxygen utilization rate in the three manures were related to their substrate condition. The low available substrate in the cow manure reduced the microbial oxygen utilization rate on Day-20. Hence, the cow manure had the largest oxygen penetration depth. Moreover, the chicken manure had a relatively higher oxygen penetration depth than did the swine manure on Day-20, although the former had the highest content of dissolved organic matters and 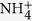/NH_3_ as the electron donor. The high 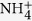/NH_3_ concentration and pH value possibly impacted the microbial activity in the chicken manure during the active stage.

Oxygen penetration depth apparently increased in the three manures during the period from Day-20 to Day-30. The moisture content decreased to 55.3% in the swine manure, 66.7% in the cow manure and 58.1% in the chicken manure on Day-30 due to water evaporation. The decrease in moisture content not only increased the porosity of the manure but also essentially limited microbial activity. It has been reported that 50% is the lower limit of the moisture^13^. The moisture content in the surface layer of the manure particles was lower than that in the core zone. Thus, the metabolic activity of the microbes was firstly limited by decreased moisture in the surface layer. Furthermore, as the amount of available substrate decreased, microbial oxygen utilization rates were significantly reduced in the three animals’ manures. Therefore, the increased porosity and decreased oxygen utilization rate greatly improved the oxygen penetration depth in the manures during the later period.

### ORP in the micro-profile of manure particles

**Figure 4:**
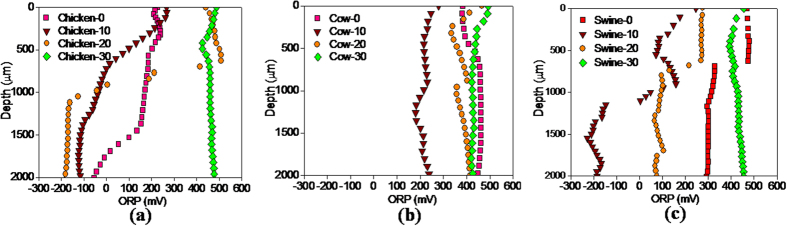
Variation of ORP with depth in the microprofiles of the manure particles of chicken (**a**), cow (**b**) and swine (**c**) on Day-0, Day-10, Day-20 and Day-30.

The ORP, as a non-biological factor, affects strongly on biological and chemical processes. The decrease in the ORP value with depth was mainly attributed to decreased DO in the three manures (Fig. 4). The cow manure had the highest ORP level among the three manures, which was in connection with it’s the lowest available substrate and 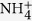/NH_3_ content as well as the largest oxygen penetration depth. It is worth noting that the ORP value in the anoxic zone of the three manures intensively decreased first and then gradually increased with composting time. During the initial 10 days, the ORP value at the 2 mm depth decreased from −50 to −120 mV in the chicken manure, from 300 to −200 mV in swine manure and from 450 to 230 mV in the cow manure. The rapid drop in the ORP in the initial period could be related to three factors: the microbial consumption of oxidizing substances, the release of reducing substances in the biodegradation process and the concentration of reducing substances in the liquid phase of the manure caused by the moisture loss. However, with the increase in oxygen penetration depth, the ORP values in the three manures all became higher than 400 mV on Day-30.

Metabolic mechanisms of organics would change with dramatic variation of the ORP level. The rapid decrease in ORP value inside the manures provided a redox potential condition for methanogenesis. That is why the CH_**4**_emission from the three animals’ manures was only detected in the initial period of composting (Fig. 2b). Furthermore, the chicken manure had the lowest ORP level and the highest CH_4_ emission rate among the three manures, and the cow manure had the highest ORP level and the lowest CH_4_ emission rate. It indicated a significant negative correlation between ORP level and CH_4_ production. As the low-ORP microenvironment gradually disappeared, the methane producing process was stopped in the later stage.

### 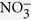 in the micro-profile of manure particles

**Figure 5:**
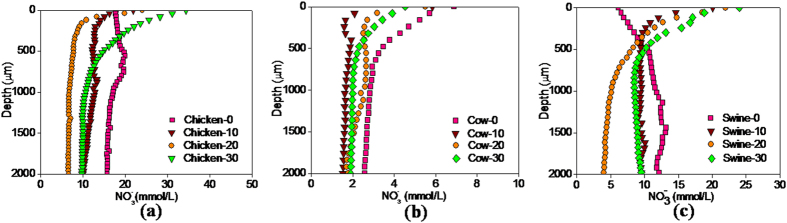
Spatial distribution of nitrate concentration with depth in the microprofile of the manure particles of chicken (**a**), cow (**b**) and swine (**c**) on Day-0, Day-10, Day-20 and Day-30.

The 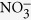 concentration in the core zone of the three manures first decreased and then gradually increased with composting time (Fig. 5a–c). During the first 20 days, the 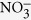 concentration in the 2 mm depth region was reduced from 15.8 to 6.6 mmol L^−1^ for the chicken manure, from 11.9 to 4.1 mmol L^−1^ for the swine manure and from 3.1 to 1.5 mmol L^−1^ for the cow manure, indicating that the 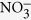 could be used as the electron acceptor by the microbes in the anoxic zone, and that the denitrification occurred in the active composting.

The profile of the 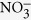 concentration shows a positive correlation between the 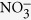 concentration and the DO level in the later stage of active composting. The 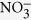 concentration in the surface of Day-30 manure particles was 4.4 times higher for swine manure, 3.5 times higher for chicken manure and 2.4 times higher for cow manure than that at 2 mm below the suface of the manure particles, respectively. This finding indicates that the nitrification process firstly occurred in the surface aerobic layer of the manure. No apparent improvement in the 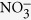 concentration was observed in the core zone of the three manures because biological nitrification would be inhibited under the anoxic and low ORP conditions. With the increase in the aerobic zone ratio in the later period, the increment of DO concentration provided the electron acceptors in the nitrification. That is why the increase in 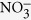 concentration was only reported in the curing stage.

## Discussion

**Figure 6:**
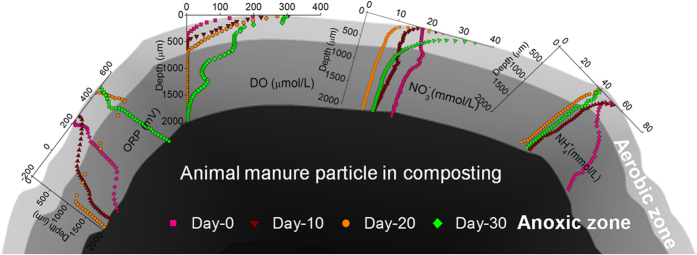
The microprofiles of DO, ORP, 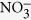 and 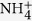
and the aerobic-anoxic dual structure in the chicken manure during active composting.

It is commonly known that organic mineralization rate under the aerobic condition is essentially faster than that under anoxic condition. The aerobic metabolism in the surface layer released more respiration heat, which increased the temperature of the composting system and substantially improved organic hydrolysis rate and anoxic metabolism efficiency in the internal zone. A portion of soluble substrate hydrolyzed in the anaerobic core possible diffused and was oxidized in the surface aerobic layer^38^. As a result, oxygen penetration was limited to the surface layer of the manure particles due to microbial oxygen utilization during the active stage. Oxygen diffusion efficiency in the manures cannot be directly improved though increasing aeration rate. Hence, the aerobic-anoxic dual structure has a long duration inside the manure particles during the active stage even under a high aeration rate (Fig. 6). The low DO level limits the organic mineralization rate in the anoxic zone. Therefore, the high ratio of anoxic zone in the manure particle could be one of the major reasons leading to the long treatment period required for active composting.

Denitrification occurred inside the manure during the initial stage due to the lack of oxygen as the electron acceptor, which was supported by the decreased concentration of nitrates. However, biological nitrification mainly occurred in the curing stage. There are several possible inhibitions for the nitrifying bacteria in the active composting stage. These are high temperature, low DO and ORP level in the manure particle, high 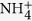/NH_3_ and available substrate content and competition among different microbial groups^24^,^25^,^26^,^27^,^28^. These factors could substantially retard the growth of nitrifying bacteria. Although the forced aeration maintained a high oxygen level in the air space of the matrix, the nitrate concentration was still quite low inside the manure during this period. With the decrease in temperature, 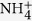/NH_3_ and dissolved organic matter concentration in the later stage, biological nitrification began in the surface layer of the manure particles due to the higher DO and ORP level. Afterwards, the 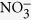 concentration significantly increased in the inner zone of the manure particles with the improvement of aerobic conditions during the curing stage.

Although low C/N ratio and high pH values are regarded as the main reasons for the nitrogen loss in the previous literature, our work suggests that ammonification, moisture loss, anoxic and high temperature conditions are important reasons leading to severe NH_3_ emission during the thermophilic stage. First, most of the proteins were hydrolyzed in the initial period and released a large amount of 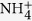/NH_3_, which increased the pH value in the liquid phase of the manure. Hence, the increased pH was the result of 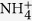/NH_3_ production rather than a cause. Second, the anoxic and high-temperature environment inhibited the biological nitrification process. The carbon sources in the organic bulking agent could not be utilized by the microbes inside the manure particles due to the space separation. Third, the high proportion of water emissions substantially concentrated the 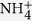/NH_3_ in the liquid phase. Meanwhile, the high-temperature condition reduced the 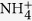/NH_3_ solubility in the water. Consequently, a large proportion of NH_3_ was released from the manure particles with water evaporation.

## Methods

### Experimental methodology

Cow, swine and chicken feces were collected from Harbin Central Red Farm in northeast China. Moisture and volatile solids contents were 75.4% and 73.9% for the swine feces, 86.7% and 86.6% for the cow feces, 79.3% and 81.2% for the chicken feces. The pH and C/N ratio were 8.1 and 8.0 for the swine feces, 8.0 and 17.7 for the cow feces, and 8.4 and 8.4 for the chicken feces, respectively. Pumice, a porous volcanic rock, was used as a bulking agent in the composting matrix to avoid the influence of organic bulking agents on the analysis of NH_3_ emission and CO_2_ production from the animal manure. The chemical and physical properties of the pumice were reported in our pervious literature^39^. Briefly, the high porosity of pumice was 7l.8–81%. The initial moisture and water-absorbing capacity of pumice were 8% and 51–72%, respectively. The volume weight and compressible strength of the pumice were 0.34–0.49 g cm^−3^ and 14–38 kg cm^−2^. The raw cow, swine and chicken feces (300 g each) were composted separately with 250 g of pumice for 30 days in three cylindrical glass reactors (5L volume, 20 cm diameter × 27 cm height). These three composting reactors were placed in a constant-temperature water bath at 55 °C for the first 20 days and at 45 °C for the next 10 days. Fresh air was blown into the composted materials from the bottom of the three reactors. To avoid the influence of the low oxygen concentration in the interparticle voids on oxygen diffusion efficiency in the manure particle, a high aeration rate of 0.2 L min^−1^ is adopted to improve air exchange and oxygen level in each composting reactor.

### Chemical analysis

The samples (2 g × 3) was dried at 105 °C and mass loss was calculated every 2 h. The moisture content of the manure samples at the different stages was determined until mass loss got inferior to 0.5% of mass loss of the previous. The volatile solids content of the dry manure samples was determined by the weight loss at 550 °C for 2 h. The pH was measured using a pH meter by dissolving 1 g manure sample in 10 mL of DI water. Manure samples were dried, ground and screened through a 200 mesh screen, and 2 g of the residues was extracted with 50 mL of DI water at 25 °C for 24 h. The aqueous extract of the manure samples was filtered through a 0.45 μm membrane. The dissolved organic carbon concentration of the aqueous extract was determined by a TOC analyzer (TOC-V_CPN_, Shimadzu). The 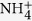/NH_3_ content in the wet manure samples was analyzed by distillation and titration using H_2_SO_4_^24^. The NH_3_ and CO_2_ releasing from the three composting reactors were absorbed by the absorption bottles filled KOH (4 mol L^−1^) and H_3_BO_3_ (1 mol L^−1^) in sequence. The NH_3_ emission rate and CO_2_ production rate of the three manures were determined by chemical titration of the solutions in the six absorption bottles every day. The CH_4_ concentration in the exhaust gas was determined three times a day by using the GC system with a flame ionization detector (6890 Series, Agilent Technologies).

### Microelectrode measurements

Four microelectrodes (DO, ORP, 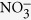 and 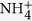) were used to investigate the chemical stratification inside the swine, cow and chicken manure particles during the composting. DO concentration profiles were obtained by using a Clark-type microelectrode with a tip diameter of 20–30 μm (OX-25, Unisense). A two-point calibration of the DO microelectrode was made with O_2_-saturated bulk water and N_2_-bubbled water. The microprofile of ORP in the particles was measure by ORP microelectrode (RD-25, Unisense) and the reference electrode (REF-25, Unisense) with a tip diameter of 20–30 μm. The ORP microelectrode was calibrated with 100 ml of pH = 4 and pH = 7 buffer solution mixed with 1 g of quinhydrone, respectively. Liquid ion-exchanging membrane microsensors for 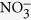 and 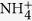 were prepared in our lab. The ionophores of 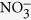 and 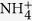 in the liquid-membrane of the microsensors were from Fluka (Sigma-aldrich, USA). The detailed manufacturing procedures were described as before^40^,^41^,^42^. The microelectrodes of 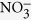 and 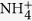 were calibrated in a dilution series (10^−2^ to 10^−6^ mol L^−1^) of 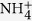
and 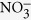 before measurement^43^,^44^.

The livestock manure samples were taken from the three composting reactors at the four different periods of Day-0, Day-10, Day-20 and Day-30. The concentrations of DO, ORP, 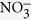 and 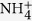 in the 2 mm depth surface layer of the manure particles were *in-situ* measured by the four microelectrodes. The four microelectrodes were combined with a computerized depth control and data acquisition (MicroProfiling System, Unisense). The microelectrodes were separately lowered into the manure particles by a computer controlled micromanipulator. All measurements were taken at spatial intervals of 20 μm through the manure particles. The microelectrodes were calibrated before each test. Spatial resolution of the microelectrodes is approximately twice the tip diameter^42^. The net oxygen fluxes in the manure particles were calculated based on the Fick’s first law of diffusion *J* = −*D*_s_d*C*/d*x* (*J*: net flux; *D*s: molecular diffusion coefficient of compound *C* in water; d*C* /d*x*: concentration gradient in the boundary layer at the surface of particle). The oxygen diffusion coefficient (2.24 × 10^5^) was used here from previous study^37^,^44^.

## Additional Information

**How to cite this article**: Wang, K. *et al.* Spatial nitrifications of microbial processes during composting of swine, cow and chicken manure. *Sci. Rep.* **5**, 14932; doi: 10.1038/srep14932 (2015).
